# Supplementary material for: Genome-Wide Identification and Expression Analysis of TCP Transcription Factors Responding to Multiple Stresses in Arachis hypogaea L
Source: Int J Mol Sci. 2025 Jan 26;26(3):1069. doi: 10.3390/ijms26031069 (PMC11816611; doi:10.3390/ijms26031069)
Supplement: Supplementary file 1 [file ijms-26-01069-s001.zip › Supplementary Table/Supplementary Table S2.docx]

Table S2 The protein sequences of the TCP gene family in *Arachis duranensis*, *Arabidopsis thaliana*, *Glycine max,* and *Arabidopsis thaliana*

| Gene ID | sequence |
| --- | --- |
| AdTCP1 | MKKDRHNKIHTSQELRDRRVRLSSEISRKFFDLQDMLEFNKPSNTLEWLFTIEAFKVKWKGGKGKLIFGRFDVKGYVTGVWKS |
| AdTCP2 | LRKSSGAGALSTTSSRSNKDRHTKVNGRGRRVRMPPLCAARIFQLTRELGHRSDGETIEWLLRHAEPSIIAATGTGTVPAAPPSASATPTMVCAGGSQWPSCRLDLCQPPPGMEYASVNGHRYMPFTSLLLQPATADESQHEEALSDQ |
| AdTCP3 | MKFSSNNTPPPHKVSINNNNNINNKDRHRKVNGRESRILLPPLCAARVFQLTRELGHKTHGDTIEWLLRHAEPSIAAATGGKSIVPPPPSSSTTNDNQGVKNGGGDTGWLEYDVLANLGVGFSANEIAVIQAMIKSFS |
| AdTCP4 | MGETQHHRPTSSRLGMRAVQGGHIVRSTGRKDRHSKVCTAKGPRDRRVRLSAHTAIQFYDVQDRLGYDRPSKAVDWLIKKAKAAIDELAELPAWKPTATTTTVAAAAAASNSNVAVQQQKLREEEENQQLNQIVHRQSTTVDDAMEASGSRKATALVGGSGRISEFQPQHLNDDNGSSGKYNSGGSGFFPPTLDTDIADTIKSFFPMVGPGEQVLFAGTTALAFDGGSSGWSEQQHHQEEQEHGRLQRMVAWNNAAADASSSGHASGFIFNSSATAAVPSPAMFGHGQFFPQRGPLQSSNTPSFRAWIDPSSIATAAMTVDHHHYLSPAAIHQASIGFAGSSSGSFSGFRIPARIQGAEEHDGVSDKPVRWLSLKMFDSEQQVKDQHWNGFSAAREFKMSLDFGCFDFNGSLNPDARLSIFSMTKLGKPK |
| AdTCP5 | MKEERKHSDSWHPTELGPKSDGQTIEWLLRQAEPSIIAATRTRTTPSSFSFVSVSVRGGAGSLSSPSSTSAASSLDHKPLLAPTPFILGKRIRTDDDASAKDDGVSVGSLVGPGAPAGLWALPARPDFDQIWSFAAAAPLLEMVVQSAMVSVSHSNKLLCSFTIGISSSNKPWEKLRRRG |
| AdTCP6 | MGGSMSSSTSSSSSSSNSTSSSTTIDANLAIATRSDDNQLQTAPKRSTKDRHTKVDGRGRRIRMPATCAARVFQLTRELGHKSDGETIEWLLQQAEPAIIAATGTGTIPANFSTLNVSLRSSGSTLSAPPSKSAPHTFHAAALALAHHHPSYEEAFHQHTALLGFHPHHAQHQHLMTADQIAEALPGGAADSSENYMRKRFREDLFKDESNHPSESQNNESGGAGAAAADGSSPKPFKTNLQMQLQQQQPQPEGGGGGGGGGGSSGHGLLRQSNLVPATAMWAVAPAPATAGAPGSTIWMLPVTAGGAGGVATGAAASEAQMWPFAHQGGGGGGGGGGGGNNTLQGPLHLMPRFNLSPSGGVEFQQEGRGGLQLGSMVMPAAQQQQQPPSQHLGLTMSESNLGMLAALNAYTRAGLNMNMNMNINSSSSHHHHHQHQPPPPPQAADSGEDGPNSSQ |
| AdTCP7 | MVLGVRSDSVVGVQDPRIVRVSRAFGGKDRHSKVCTIRGLRDRRVRLSVPTAIQLYDLQDRLGLNQPSKVVDWLLNAAKHEIDELPPLPHINNPFTNLSGFPSSSSNSMINTNEASTSMFNINRSIHQWQGLAQNYSKWNNNKSKSSSSTKQASSEDNNQMVC |
| AdTCP8 | MTNLAVADAMFKQEYFTAAAAGGSSHHHHHHYGMSSLITKRPAPKKDRHSKIHTSQGLRDRRVRLSIEIARKFFDLQDMLGFDKASNTLEWLLTKSKRAIKELARSKNSSSGNNNGGVANNSFSSSSSDGADDDEVDSVINQQQLTDNIDGTHEQGGVDSLERRLRRDQQKEEESRAKMKESREKARARARERTSSKMCNSEKVQDLKKKSPAMVMEEDPQILMQHQQLRAPMIQPLEESSMRSSPKLFQPHHLAAAAGGGGGGGSSEVPRDDFNVIEESIVIRRKLKQSLMSSSSSSSNNNHHQNHNVIPKEATNFNNNNSDYHCFPNLSSPNWEANASAATNNGRSNVCTISSMNLSTMFCFLLLF |
| AdTCP9 | MGMKSTGGEIVQVQGGHIVRSTGRKDRHSKVYTAKGPRDRRVRLSAHTAIQFYDVQDRLGYDRPSKAVDWLIKKAKAAIDKLAELPPWEPTPPPPTTNEVEEEQNGGSTDMAIAEQSESSGYNFQLQRQLGEDPENQHHHSAFIPSPIDTDGGIAFFPTTSAASSINFQSYPPEIISRTNNSSEDLGLSLHSFQDSGLIHHHGQSQQGGGADHQNPSSNDQTLFANYQRMVAWNSDAGGTQADMNRSGFMVNSPGFSSAFSHHQQRGTLQSSFSPSLRPWSEIPQMASSNEHHHNHKSQPIQQQHQASIGEDESHGVGSDRPSSSASPNSHH |
| AdTCP10 | MVPSLLSGTMELEPAANQFRVKNKFDEAVVKKARRMKEIESSSSDLAAGLDPRIVRVSRALGGKDRHSKVCTIRGLRDRRVRLSVPTAINLYDLQDRLGLNQPSKVVDWLLNAAKHEIDDLPPLPIPPSNFTLACYPSLPTNKSNHKEQDSSNNNTSAHVLPNTFLLPTTINHNHHPPSFLGLLNTMPSSIDLHGGVNVVNLPFQNLGASQILCCSPSPLQRAAAAATTTVTTTQSYFSPSSLSHYNNDNVAAAMEIMDPRQINHHHQMMMVMSNHQNLATSPNRSESQSHADDSAN |
| AdTCP11 | MFPSTTYSYSTGPYPCFPSSSSSYPLFPFLNPENNASSSSATTATNNNNNNNLLHDPSLCVPYIPIPEALTNLATVVDNTTNLAASSSMPKQQDLNGGGGGGAHHHFGISSLLTKKPAAAKKDRHSKIYTAQGLRDRRVRLSIEIARKFFDLQDMLGFDKASNTLDWLFTKSKKAIKELARSKNHSASDEAEAGAGAGANNKSFASSSDCDENDFEVLSRNNHHRHQQGLVRSDARERKLKNAQQKEASACNVRAKMKESREKARARARERTSNKMCNSIMGNSSSSSGKVLELKKRCPAPSENPHHLGGAEVSQTRDDFNVIEESIVIKRKLKQPSMMSSSHHHHHHHHQLNLPIPNNNKEASFNNNSDNYHPGNYTNLSPNWDNANGPASTANNRSNFCAIASMNLSTGLQIFGKSWEECTNPSRLH |
| AiTCP1 | KDRHSKIHISHGLRDRRVRLSSEIVRKTCWSSTSPATRSNGFSPVKEEAHQLAQIFETVGAFKDSTLYHFSMFHASHHIQPVPAIKEEVEGIGLLRAIELILLFGLIRFFLVRSFDVKGYVTGVWES |
| AiTCP2 | MKFSSNNTPPPRKVSINNNNNNKDRHKKVNGRESRILLPPLCAARVFQLTRELGHKTHGDTIEWLLRHAEPSIAAATSGKSIVPPPSSSSNTNDNNGNQGMKNGGGDTGWLEYDVLANLGVGFSANEIAVIQAMIKSFS |
| AiTCP3 | MGMRSNSNKGGEIIQVEGGHIVRSTGRKDRHSKVYTAKGPRDRRVRLSAHTAIEFYDVQDRLGYDRPSKAQFLQIQQQQQQNLETDPIAFFPTSSATPSMNFQNYPHQMISRTANNNVNPITEDLGLSLHPFQQAPSNQHQGTLFGAGSSNNVGFESQYQKIVAWNNEAATMDINRVGFMVNPQQNHHHPFLGQSVSGSLQSSFPPPSLRSWGEIQMNPNSSEQQHSRPQQLIHHASIFGSRFVSDGLAAGFCIPARIQGDQENHHGVASNTTPSSASPHSHH |
| AiTCP4 | MGETQHHRPTSSRLGMRAVQGGHIVRSTGRKDRHSKVCTAKGPRDRRVRLSAHTAIQFYDVQDRLGYDRPSKAVDWLIKKAKAAIDELAELPAWKPTATTTTVAAAAAAASNSNVAVQQQKLREEEENQQLNQIVHRQSTTVDDAMEASGSRKATALVGGSGRISEFQPQHLNDDNGSSGKYNSGGSGFFPPTLDTDIADTIKSFFPMVGPGEVATTTSSFHNYPPPPDLLSLLFAGTTALAFDGGSSGWSEQQHHQEEQEHGRLQRMVAWNNAAADASSSGHASGFIFNSSATAAVPSPAMFGHGQFFPQRGPLQSSNTPSFRAWIDPSSIATAAMTVDHHHYLSPAAIHQASIGFAGSSSGSFSGFRIPARIQGAEEHDGMFDSEQQVKDQHWNGFSAAREFKMSLDFGCFDFNGSLSMPWE |
| AiTCP5 | MGGSISSSSSSSSSSSNSTSSSTTIDANLAIATRSDDNQLQTAPKRSTKDRHTKVDGRGRRIRMPATCAARVFQLTRELGHKSDGETIEWLLQQAEPAIIAATGTGTIPANFSTLNVSLRSSGSTLSAPPSKSAPHTFHAAALALAHHHPSYEEAFHPHTALLGFHPHHAQHQHLMTADQIAEALPGGAADSSENYMRKRFREDLFKDESNHQSESQNNESGGTGAVAADGSSPKPFKTNLQMQLQQQQPQPEGGGAGGGGGSSGHGLLRQSNLVPATAMWAVAPAPATAGAPGSTIWMLPVTAGGAGGVATGAAASEAQMWPFAHQGGSGGGGGGGNNTLQGPLHLMPRFNLSPSGGVEFQQEGRGGLQLGSMVMPAAQQQQQPPSQHLGLTMSESNLGMLAALNAYTRAGLNMNMNMNINSSSSHHHHHHHHQPPPPPPQAADSGEDGPNSSQ |
| AiTCP6 | MTNLAVADAMFKQEYFTAGGGGGGGGSSHHHHHHYGMSSLITKRPAPKKDRHSKIHTSQGLRDRRVRLSIEIARKFFDLQDMLGFDKASNTLEWLLTKSKRAIKELARSKNSSSGNNNGGVANNNSFSSSSSDGVDDDEVDSVINQQQLTDNIDGTHEQGGVDSLERRLRRDQQKDEESRAKMKESREKARARARERTSSKMCNSEKVQELKKKSPAMVMEEDPQILMQHQQLRAPMIQPLEESSMRSSPKLFQPHHLAAAGGGGGGGGGGGGGSSEVPRDDFNVIEESIVIRRKLKQSLMSSSSSSSNNHHHQNHNVIPKEATNFNNNNSNNSDYHCFPNLSSPNWESNASAATNNGRSNVCTISSMNLSTRLQIFGKSWEECTNPHL |
| AiTCP7 | MGMKSTGGEIVQVQGGHIVRSTGRKDRHSKVYTAKGPRDRRVRLSAHTAIQFYDVQDRLGYDRPSKAVDWLIKKAKAAIDKLAELPPWEPTPPPPTTNEVEEDQNGGSTDMAIAEQSESSGYNFQLQRQLGEDPENQHHHSAFIPSPIDTDGGIAFFPTTSAASSINFQSYPPEIISRTNNSSEDLGLSLHSFQDSGLIHHHGQSQQGGGADHQNPSSNDQTLFANYQRMVAWNSDAGGTQADMNRSGFMVNSPGFSSAFSHHQQRGTLQSSFSPSLRPWSEIPQMASSNEHHSHGVGSDRPSSSASPNSHH |
| AiTCP8 | MMMETKEEGMIIEENKKKSNENDKFTLKGSAAASSSTRQWSAFRNPRIVRVSRSFGGKDRHSKVCTIRGLRDRRIRLSVPTAIQLYDLQDKLGLSQPSKVIDWLLDATKLDIDKLPPLHFPQPCFQPPQTLFPHLLHDPSSFWKPRFWELDSSASSSSSSRFLKGKDLMTTMPSDNNNNKAKWIFKSNNADHENQEFGGGYTTQRLFPMETQTTTNNNPFQMGDYYGSYPMMNNGGLMQLNHLSSLQVGPSGSSQL |
| AiTCP9 | MTLLSSIKRNIASIEKKREDNYIKNESMQQETSLYLIAIPIHKNNCAQPLFEMCNKWSLHWYYGVGTSCKSIQSQKQLETKKNERDRFIIIRSCSWPGFKNNKKLGLNQPSKVVDWLLNAAKHEIDDLPPLPIPPSNFTLACYPSLPTNKSNHNKEQDSSNNTSAHVLPNSFLLPTTINHNHHPPSFLGLLNTMPSSIGASQILCCSPSPLPRAAAAATTATTQSYFSPSSFSHYNNNNVAAAMEIMDPRQINHHHHGDESSSESCKIT |
| AiTCP10 | MFPSTTYSYSTGPYPCFPSSSSSYPLFPFLNPENNASSSSATTATNNNNNNLLHDPSLCVPYIPIPEALTNLATVVDNTSNLAASSSMPKQHDLNGGGGGGAHHHFGISSLLAKKPAAAKKDRHSKIYTAQGLRDRRVRLSIEIARKFFDLQDMLGFDKASNTLDWLFTKSKKAIKELARSKNHSASDEAEAGAGAGANNKSFASSSDCDEDDFEVLSRNNHHRHHQQGLVWSDARERKLKSAQQKEASACNVRAKMKESREKARARARERTSNKMCNSIMGNSSSSSGKVLELKKRCPAPSENPHHLGGGEVSQTRDDFNVIEESIVIKRKLKQPSMMSSSHHHHHHHHQLNLPIPNNNNKEASFNNNSDNNYHPGNYTNLSPNWDNANGPASSANNRSNFCAIASMNLSTGMHVFLVRNRNCGC |
| AtTCP1 | MSSSTNDYNDGNNNGVYPLSLYLSSLSGHQDIIHNPYNHQLKASPGHMVSAVPESLIDYMAFKSNNVVNQQGFEFPEVSKEIKKVVKKDRHSKIQTAQGIRDRRVRLSIGIARQFFDLQDMLGFDKASKTLDWLLKKSRKAIKEVVQAKNLNNDDEDFGNIGGDVEQEEEKEEDDNGDKSFVYGLSPGYGEEEVVCEATKAGIRKKKSELRNISSKGLGAKARGKAKERTKEMMAYDNPETASDITQSEIMDPFKRSIVFNEGEDMTHLFYKEPIEEFDNQESILTNMTLPTKMGQSYNQNNGILMLVDQSSSSNYNTFLPQNLDYSYDQNPFHDQTLYVVTDKNFPKGKVWIQDSFVN |
| AtTCP2 | MIGDLMKNNNNGDVVDNEVNNRLSRWHHNSSRIIRVSRASGGKDRHSKVLTSKGPRDRRVRLSVSTALQFYDLQDRLGYDQPSKAVEWLIKAAEDSISELPSLNNTHFPTDDENHQNQTLTTVAANSLSKSACSSNSDTSKNSSGLSLSRSELRDKARERARERTAKETKERDHNHTSFTDLLNSGSDPVNSNRQWMASAPSSSPMEYFSSGLILGSGQQTHFPISTNSHPFSSISDHHHHHPHHQHQEFSFVPDHLISPAESNGGAFNLDFNMSTPSGAGAAVSAASGGGFSGFNRGTLQSNSTNQHQSFLANLQRFPTSESGGGPQFLFGALPAENHHHNHQFQLYYENGCRNSSEHKGKGKN |
| AtTCP3 | MAPDNDHFLDSPSPPLLEMRHHQSATENGGGCGEIVEVQGGHIVRSTGRKDRHSKVCTAKGPRDRRVRLSAPTAIQFYDVQDRLGFDRPSKAVDWLITKAKSAIDDLAQLPPWNPADTLRQHAAAAANAKPRKTKTLISPPPPQPEETEHHRIGEEEDNESSFLPASMDSDSIADTIKSFFPVASTQQSYHHQPPSRGNTQNQDLLRLSLQSFQNGPPFPNQTEPALFSGQSNNQLAFDSSTASWEQSHQSPEFGKIQRLVSWNNVGAAESAGSTGGFVFASPSSLHPVYSQSQLLSQRGPLQSINTPMIRAWFDPHHHHHHHQQSMTTDDLHHHHPYHIPPGIHQSAIPGIAFASSGEFSGFRIPARFQGEQEEHGGDNKPSSASSDSRH |
| AtTCP4 | MSDDQFHHPPPPSSMRHRSTSDAADGGCGEIVEVQGGHIVRSTGRKDRHSKVCTAKGPRDRRVRLSAHTAIQFYDVQDRLGFDRPSKAVDWLIKKAKTSIDELAELPPWNPADAIRLAAANAKPRRTTAKTQISPSPPPPQQQQQQQQLQFGVGFNGGGAEHPSNNESSFLPPSMDSDSIADTIKSFFPVIGSSTEAPSNHNLMHNYHHQHPPDLLSRTNSQNQDLRLSLQSFPDGPPSLLHHQHHHHTSASASEPTLFYGQSNPLGFDTSSWEQQSSEFGRIQRLVAWNSGGGGGATDTGNGGGFLFAPPTPSTTSFQPVLGQSQQLYSQRGPLQSSYSPMIRAWFDPHHHHQSISTDDLNHHHHLPPPVHQSAIPGIGFASGEFSSGFRIPARFQGQEEEQHDGLTHKPSSASSISRH |
| AtTCP5 | MRSGECDEEEIQAKQERDQNQNHQVNLNHMLQQQQPSSVSSSRQWTSAFRNPRIVRVSRTFGGKDRHSKVCTVRGLRDRRIRLSVPTAIQLYDLQDRLGLSQPSKVIDWLLEAAKDDVDKLPPLQFPHGFNQMYPNLIFGNSGFGESPSSTTSTTFPGTNLGFLENWDLGGSSRTRARLTDTTTTQRESFDLDKGKWIKNDENSNQDHQGFNTNHQQQFPLTNPYNNTSAYYNLGHLQQSLDQSGNNVTVAISNVAANNNNNLNLHPPSSSAGDGSQLFFGPTPPAMSSLFPTYPSFLGASHHHHVVDGAGHLQLFSSNSNTASQQHMMPGNTSLIRPFHHLMSSNHDTDHHSSDNESDS |
| AtTCP6 | MVMEPKKNQNLPSFLNPSRQNQDNDKKRKQTEVKGFDIVVGEKRKKKENEEEDQEIQILYEKEKKKPNKDRHLKVEGRGRRVRLPPLCAARIYQLTKELGHKSDGETLEWLLQHAEPSILSATVNGIKPTESVVSQPPLTADLMICHSVEEASRTQMEANGLWRNETGQTIGGFDLNYGIGFDFNGVPEIGFGDNQTPGLELRLSQVGVLNPQVFQQMGKEQFRVLHHHSHEDQQQSAEENGS |
| AtTCP7 | MSINNNNNNNNNNNDGLMISSNGALIEQQPSVVVKKPPAKDRHSKVDGRGRRIRMPIICAARVFQLTRELGHKSDGQTIEWLLRQAEPSIIAATGTGTTPASFSTASVSIRGATNSTSLDHKPTSLLGGTSPFILGKRVRADEDSNNSHNHSSVGKDETFTTTPAGFWAVPARPDFGQVWSFAGAPQEMFLQQQHHHQQPLFVHQQQQQQAAMGEASAARVGNYLPGHLNLLASLSGGSPGSDRREEDPR |
| AtTCP8 | MDLSDIRNNNNDTAAVATGGGARQLVDASLSIVPRSTPPEDSTLATTSSTATATTTKRSTKDRHTKVDGRGRRIRMPALCAARVFQLTRELGHKSDGETIEWLLQQAEPAIVAATGTGTIPANFSTLSVSLRSSGSTLSAPPSKSVPLYGALGLTHHQYDEQGGGGVFAAHTSPLLGFHHQLQHHQNQNQNQDPVETIPEGENFSRKRYRSVDLSKENDDRKQNENKSLKESETSGPTAAPMWAVAPPSRSGAGNTFWMLPVPTTAGNQMESSSNNNTAAGHRAPPMWPFVNSAGGGAGGGGGAATHFMAGTGFSFPMDQYRGSPLQLGSFLAQPQPTQNLGLSMPDSNLGMLAALNSAYSRGGNANANAEQANNAVEHQEKQQQSDHDDDSREENSNSSE |
| AtTCP9 | MATIQKLEEVAGKDQTLRAVDLTIINGVRNVETSRPFQVNPTVSLEPKAEPVMPSFSMSLAPPSSTGPPLKRASTKDRHTKVEGRGRRIRMPATCAARIFQLTRELGHKSDGETIRWLLENAEPAIIAATGTGTVPAIAMSVNGTLKIPTTTNADSDMGENLMKKKRKRPSNSEYIDISDAVSASSGLAPIATTTTIQPPQALASSTVAQQLLPQGMYPMWAIPSNAMIPTVGAFFLIPQIAGPSNQPQLLAFPAAAASPSSYVAAVQQASTMARPPPLQVVPSSGFVSVSDVSGSNLSRATSVMAPSSSSGVTTGSSSSIATTTTHTLRDFSLEIYEKQELHQFMSTTTARSSNH |
| AtTCP10 | MGLKGYSVGEGGGEIVEVQGGHIIRATGRKDRHSKVFTSKGPRDRRVRLSAHTAIQFYDVQDRLGYDRPSKAVDWLIKKAKTAIDKLELGETTTTTTRQEPVNTKPESPTLVFQRENNDQTQFVAANLDPEDAMKTFFPATTTTNGGGGTNINFQNYPHQDDNNMVSRTTTPPPNLSQDLGLSLHPFQGNNNTVVVPETNNFTTTHFDTFGRISGWNHHDLTMTSSSSSEHQQQEQEERSNGGFMVNHHPHHHHHQPSMMTLLNSQQQQVFLGGQQQQQQRGTLQSSLFPHSFRSWDHHQTTSDHHHHQNQASSMFASSSQYGSHGMMMMQGLSFPNTTRLLHGEEATQPNSSSSPPNSHL |
| AtTCP11 | MIFQNVCRNESNFNAIASESRSQTQFGVSKSSSSGGGCISARTKDRHTKVNGRSRRVTMPALAAARIFQLTRELGHKTEGETIEWLLSQAEPSIIAATGYGTKLISNWVDVAADDSSSSSSMTSPQTQTQTPQSPSCRLDLCQPIGIQYPVNGYSHMPFTAMLLEPMTTTAESEVEIAEEEERRRRHH |
| AtTCP12 | MFPSLDTNGYDLFDPFIPHQTTMFPSFITHIQSPNSHHHYSSPSFPFSSDFLESFDESFLINQFLLQQQDVAANVVESPWKFCKKLELKKKNEKCVDGSTSQEVQWRRTVKKRDRHSKICTAQGPRDRRMRLSLQIARKFFDLQDMLGFDKASKTIEWLFSKSKTSIKQLKERVAASEGGGKDEHLQVDEKEKDETLKLRVSKRRTKTMESSFKTKESRERARKRARERTMAKMKMRLFETSETISDPHQETREIKITNGVQLLEKENKEQEWSNTNDVHMVEYQMDSVSIIEKFLGLTSDSSSSSIFGDSEECYTSLSSVRGMSTPREHNTTSIATVDEEKSPISSFSLYDYLCY |
| AtTCP13 | MNIVSWKDANDEVAGGATTRREREVKEDQEETEVRATSGKTVIKKQPTSISSSSSSWMKSKDPRIVRVSRAFGGKDRHSKVCTLRGLRDRRVRLSVPTAIQLYDLQERLGVDQPSKAVDWLLDAAKEEIDELPPLPISPENFSIFNHHQSFLNLGQRPGQDPTQLGFKINGCVQKSTTTSREENDREKGENDVVYTNNHHVGSYGTYHNLEHHHHHHQHLSLQADYHSHQLHSLVPFPSQILVCPMTTSPTTTTIQSLFPSSSSAGSGTMETLDPRQM |
| AtTCP14 | MQKPTSSILNVIMDGGDSVGGGGGDDHHRHLHHHHRPTFPFQLLGKHDPDDNHQQQPSPSSSSSLFSLHQHQQLSQSQPQSQSQKSQPQTTQKELLQTQEESAVVAAKKPPLKRASTKDRHTKVDGRGRRIRMPALCAARVFQLTRELGHKSDGETIEWLLQQAEPSVIAATGTGTIPANFTSLNISLRSSGSSMSLPSHFRSAASTFSPNNIFSPAMLQQQQQQQRGGGVGFHHPHLQGRAPTSSLFPGIDNFTPTTSFLNFHNPTKQEGDQDSEELNSEKKRRIQTTSDLHQQQQQHQHDQIGGYTLQSSNSGSTATAAAAQQIPGNFWMVAAAAAAGGGGGNNNQTGGLMTASIGTGGGGGEPVWTFPSINTAAAALYRSGVSGVPSGAVSSGLHFMNFAAPMAFLTGQQQLATTSNHEINEDSNNNEGGRSDGGGDHHNTQRHHHHQQQHHHNILSGLNQYGRQVSGDSQASGSLGGGDEEDQQD |
| AtTCP15 | MDPDPDHNHRPNFPLQLLDSSTSSSSTSLAIISTTSEPNSEPKKPPPKRTSTKDRHTKVEGRGRRIRMPAMCAARVFQLTRELGHKSDGETIEWLLQQAEPAVIAATGTGTIPANFTSLNISLRSSRSSLSAAHLRTTPSSYYFHSPHQSMTHHLQHQHQVRPKNESHSSSSSSSQLLDHNQMGNYLVQSTAGSLPTSQSPATAPFWSSGDNTQNLWAFNINPHHSGVVAGDVYNPNSGGSGGGSGVHLMNFAAPIALFSGQPLASGYGGGGGGGGEHSHYGVLAALNAAYRPVAETGNHNNNQQNRDGDHHHNHQEDGSTSHHS |
| AtTCP16 | MDSKNGINNSQKARRTPKDRHLKIGGRDRRIRIPPSVAPQLFRLTKELGFKTDGETVSWLLQNAEPAIFAATGHGVTTTSNEDIQPNRNFPSYTFNGDNISNNVFPCTVVNTGHRQMVFPVSTMTDHAPSTNYSTISDNYNSTFNGNATASDTTSAATTTATTTV |
| AtTCP17 | MGIKKEDQKSSLSLLTQRWNNPRIVRVSRAFGGKDRHSKVCTVRGLRDRRIRLSVMTAIQVYDLQERLGLSQPSKVIDWLLEVAKNDVDLLPPLQFPPGFHQLNPNLTGLGESFPGVFDLGRTQREALDLEKRKWVNLDHVFDHIDHHNHFSNSIQSNKLYFPTITSSSSSYHYNLGHLQQSLLDQSGNVTVAFSNNYNNNNLNPPAAETMSSLFPTRYPSFLGGGQLQLFSSTSSQPDHIE |
| AtTCP18 | MNNNIFSTTTTINDDYMLFPYNDHYSSQPLLPFSPSSSINDILIHSTSNTSNNHLDHHHQFQQPSPFSHFEFAPDCALLTSFHPENNGHDDNQTIPNDNHHPSLHFPLNNTIVEQPTEPSETINLIEDSQRISTSQDPKMKKAKKPSRTDRHSKIKTAKGTRDRRMRLSLDVAKELFGLQDMLGFDKASKTVEWLLTQAKPEIIKIATTLSHHGCFSSGDESHIRPVLGSMDTSSDLCELASMWTVDDRGSNTNTTETRGNKVDGRSMRGKRKRPEPRTPILKKLSKEERAKARERAKGRTMEKMMMKMKGRSQLVKVVEEDAHDHGEIIKNNNRSQVNRSSFEMTHCEDKIEELCKNDRFAVCNEFIMNKKDHISNESYDLVNYKPNSSFPVINHHRSQGAANSIEQHQFTDLHYSFGAKPRDLMHNYQNMY |
| AtTCP19 | MESNHEGNAIQVIDQVTTMTHLSDPNPKTKPGMMLMKQEDGYLQPVKTKPAPKRPTSKDRHTKVEGRGRRIRMPAGCAARVFQLTRELGHKSDGETIRWLLERAEPAIIEATGTGTVPAIAVSVNGTLKIPTSSPVLNDGGRDGDGDLIKKRRKRNCTSDFVDVNDSCHSSVTSGLAPITASNYGVNILNVNTQGFVPFWPMGMGTAFVTGGPDQMGQMWAIPTVATAPFLNVGARPVSSYVSNASDAEAEMETSGGGTTQPLRDFSLEIYDKRELQFLGGSGNSSPSSCHET |
| AtTCP20 | MDPKNLNRHQVPNFLNPPPPPRNQGLVDDDAASAVVSDENRKPTTEIKDFQIVVSASDKEPNKKSQNQNQLGPKRSSNKDRHTKVEGRGRRIRMPALCAARIFQLTRELGHKSDGETIQWLLQQAEPSIIAATGSGTIPASALASSAATSNHHQGGSLTAGLMISHDLDGGSSSSGRPLNWGIGGGEGVSRSSLPTGLWPNVAGFGSGVPTTGLMSEGAGYRIGFPGFDFPGVGHMSFASILGGNHNQMPGLELGLSQEGNVGVLNPQSFTQIYQQMGQAQAQAQGRVLHHMHHNHEEHQQESGEKDDSQGSGR |
| AtTCP21 | MADNDGAVSNGIIVEQTSNKGPLNAVKKPPSKDRHSKVDGRGRRIRMPIICAARVFQLTRELGHKSDGQTIEWLLRQAEPSIIAATGTGTTPASFSTASLSTSSPFTLGKRVVRAEEGESGGGGGGGLTVGHTMGTSLMGGGGSGGFWAVPARPDFGQVWSFATGAPPEMVFAQQQQPATLFVRHQQQQQASAAAAAAMGEASAARVGNYLPGHHLNLLASLSGGANGSGRREDDHEPR |
| AtTCP22 | MNQNSSVAEATLQLNSGEKPSPGSIPFISSGQHGNISTSATSSTSTSSGSALAVVKSAVKKPTKDRHTKVDGRGRRIRMPAMCAARVFQLTRELGHKSDGETIEWLLQQAEPAIIASTGTGTIPANFSTLNASLRSGGGSTLFSQASKSSSSPLSFHSTGMSLYEDNNGTNGSSVDPSRKLLNSAANAAVFGFHHQMYPPIMSTERNPNTLVKPYREDYFKEPSSAAEPSESSQKASQFQEQELAQGRGTANVVPQPMWAVAPGTTNGGSAFWMLPMSGSGGREQMQQQPGHQMWAFNPGNYPVGTGRVVTAPMGSMMLGGQQLGLGVAEGNMAAAMRGSRGDGLAMTLDQHQHQLQHQEPNQSQASENGGDDKK |
| AtTCP23 | MESHNNNQSNNNTTGSAHLVPSMGPISGSVSLTTTAPNSTTTTVTAAKTPAKRPSKDRHIKVDGRGRRIRMPAICAARVFQLTRELQHKSDGETIEWLLQQAEPAIIAATGTGTIPANISTLNISLRSSGSTLSAPLSKSFHMGRAAQNAAVFGFQQQLYHPHHITTDSSSSSLPKTFREEDLFKDPNFLDQEPGSRSPKPGSEAPDQDPGSTRSRTQNMIPPMWALAPTPASTNGGSAFWMLPVGGGGGPANVQDPSQHMWAFNPGHYPGRIGSVQLGSMLVGGQQLGLGVAENNNLGLFSGGGGDGGRVGLGMSLEQKPQHQVSDHATRDQNPTIDGSP |
| AtTCP24 | MEVDEDIELQKHQEQQSRKLQRFSEDNTGLMRNWNNPSSRIIRVSRASGGKDRHSKVLTSKGLRDRRIRLSVATAIQFYDLQDRLGFDQPSKAVEWLINAASDSITDLPLLNTNFDHLDQNQNQTKSACSSGTSESSLLSLSRTEIRGKARERARERTAKDRDKDLQNAHSSFTQLLTGGFDQQPSNRNWTGGSDCFNPVQLQIPNSSSQEPMNHPFSFVPDYNFGISSSSSAINGGYSSRGTLQSNSQSLFLNNNNNITQRSSISSSSSSSSPMDSQSISFFMATPPPLDHHNHQLPETFDGRLYLYYGEGNRSSDDKAKERR |
| GmTCP1 | MELEADHQNGIRSRPNFPLQLLEKKDVDDVTEQPCSTTSVVTTAAATITSGNNDLHVAEQSKKPPPKRSSTKDRHTKVDGRGRRIRMPAACAARVFQLTRELGHKSDGETIEWLLQQAEPAVIAATGTGTIPANFTSLNISLRSSGSTMSAPSHYFRGNYFNPSTFSTSAAAASAAAVAQLRSRAEWDRNMSMVMEDSRRSMLENTSSISAILNFNPMGNVNVIQQAKQELREESAAAGGGGSLDSVASDSDGSLGRKRRPEQELSQMGSYLIQSSTGSLPSSHASNTAAFWMVAGHGNQAMSGGGGSDGSSDNPIWAIPSVGNSGVYRGAMSAPGGIHFMNFASPMNLIPGAQLGSAMVGGGSGGGNSGSQLLSESNLGSACGP |
| GmTCP2 | MELEADHQNGIRSRLNFPLQLLEKKDIDDVTEQPCSTTSVVTATTTAITTTTTSDNNDLHLAEQSKKPPPKRSSTKDRHTKVDGRGRRIRMPAACAARVFQLTRELGHKSDGETIEWLLQQAEPAVIAATGTGTIPANFTSLNISLRSSGSTMSAPSHYFRGNYFNPSTFSTSAAAAAAAQLRSRAEWDRSMSMVMEDSRRSMLENTSSISAILNFNPMGNVNVIQQAKQELREESGGGGGLESVASDSDGSLGRKRRPEQELSQMGSYLIQSSTGSLPASHASNTAAFWMVAGHGNQAMSGGGSGGGGNNGSSDNNPIWAIPSVGNSGVYRGAMSAPGGIHFMNFASPMNLMPGGQLGSGIVGGGGGGGGNGGAQLLSESNLGMLAALNAYRQIPANGVSEPPGSAGQHHGGDDGRDSTSQHS |
| GmTCP3 | MADEASGDVDLRLGEPKGPKEEPETEEGSAIRVMPVAVHVPAGMSMSMSKALAQAQVQAQPQKRASIKDRHTKVEGRGRRIRMPATCAARIFQLTRELGHKSDGETIRWLLEHAEPAIIAATGTGTVPAIAMSVNGTLKIPTTSPSDQEPGEPPERKKRKRPANSAYVDINGAAVSVSAGLASSIINNNNQNAATTMTTTMAIPQHTAIPLPQGMVPVWAIPSNAVVPAAGAFFVVPQTASFQHQPQFFTIARPISEFVSSMVTPVVQLQPTSINSSTNTNSNASSTAPSSKSPARATVMAPTTTTTTTRQMLRDFSLEIYDKQELQFMSRASSKH |
| GmTCP4 | MQSQSSDTFPSHNGNDPISCYDPAFYYYNSRPFSPIDSESNPTSKQDNNNNCDLVPSPTPLSFFHFPSSPFEDTQNQILLEQHHDFLLQFHHQSLPKDPVPQPAVITTMDPFVKKSDQIQRKRPGKRDRHSKINTARGLRDRRMRLSLEVAKRFFGLQDMLNFDKASKTVEWLLNQAKVEINRLVKEKKKNDHHHQSCSSASSECEEGVSSLDEVVVSRDQEQQQQQQQEKVEKVVKRRVKNSRKISAFDPLAKECRERARERARERTREKMRSRGVLAEESKQCGEETNQDLIQLGSSNPFETGDQESGAKTSHSVDVHPSSLDVIATEAKEQSYRAVKEHNDDDDDSLVVLSKWSPSLIFNNSGFSQDHQFAEFQSLGKPWET |
| GmTCP5 | MIMMMASSREGRQAKQEGDTTNIDKFSQASSTSRQWSAFRNPRIVRVSRSFGGKDRHSKVCTIRGLRDRRIRLSVPTAIQLYDLQDKLGLSQPSKVIDWLLEATKFDIDKLPPLQFPQGFGQFQHPQTLLPFHDSSAASHQLSLGPFCDASSTFVRDGGIQNLMAKSRYWENIDSMSRLRGNKEAEKGKWIKTSEQENHDQDGVVGGYNSLQVSTQRLFPMRSSSTTTTTTHSLLPGLFNNSMPYNSYNSEPSSLSLSQFGSHGLFPTQQVDPNPSSGNNGVQFQSSFPLPSSGSQLFFGSSSATPSMFTTYAPFIAPSVDTDPRQFNHIQFLNSGSHILPHPPLIPSLHHSFNSHVRPFPEAPFSSKLLDSNSSNRSQENHKGSTS |
| GmTCP6 | MKGGSDDFHHHHHHHHHNHHRQNFPFQLLEKKEDQQDPSSCSTSSPYPPLAILAEPSTSNNNNQLLPTPSSEPSPKKPPPKRTSTKDRHTKVDGRGRRIRMPALCAARVFQLTRELGHKSDGETIEWLLQQAEPSVIAATGTGTIPANFTSLNISLRSSGSSMSVPSQLRSSYFHNSNFSLQPRRSLFLENTNTPPTTTTLLHFQSNNNNAIFQPKHELRESSSLDLSSENNNEEGNFGRKRRPLEQDLSSTIQHQVGNYLMQSNTSGTIPASHHAQIPANIWMLANSNNHHLGGGVSGEAPPLWTFPGVSNSAALYRGTMSSGLHFMNFPTPMALLPGQQLGSSGIGGNMNNNEGHLSMLSGLNPNYRTTVIGISESQASESQSTHHGGSEDRHEHE |
| GmTCP10 | MYSSNTSLNGNELISYPNQPFCFRPFSFESNPTNSSKEETNSNYALPLPPPPPLLSFFQSPFDENIFQEHHHDFLLLHHSLADSGVLTKNLDVAAEISPIPCPGEGSIAMEHTPRKRSSKRDRHSKINTARGLRDRRMRLSLEVAKRFFGLQDMLGFDKASKTVDWLLNQAKGEIKQLGREKTSVGGAKSASSTSECEGVSSLDEVVVSGGVNEEQERETVPNMKRRTNKVCRKSAFNPIDKESREKARERARERTREKMRTRRVLADASNLNRLSSWNPFETVEDSAGTTHQSQSVNHPSLDVHLPEADQEPSSHNAKEHWGENMAHEDNSLAIMNKWSPTMMFNSLHNSGVLQEHQFAEFQSLGKPWEAYSNHI |
| GmTCP7 | MMIESSGKVHEAKNQQEGDDDNNIKIEKLLKARPSTSSSSRSWSAFRNPRIVRVSRSLGSKDRHSKVCTIRGLRDRRIRLSVPTAIQLYNLQDKLGFNQPSKVVDWLLEATKSDIDKLPPLQFPHCFAHQFHQQTLLPGTSQFSLGGFYDAANNNSTFIKDGGNQNRLTATKSTRYWDIDSLNNGKELAESVSISQKGQFWIKTNEQENHQGGGIGGSSTTTHNREDNSSVHYKLFPIGTTTNNSYLPGLLNNGMTHNSYHHSEPSRLSLSHFGSHGLFPSHDSHQSSGIGVPFSSSNFSGASSGPQLLFCPTSATPSAFYSVESDPRQSNNNVQIFSSSSQVMKRLPLIQSLHSTNSPISRRLPTSFSSKLLDSDNNDRSQPNKGTSSRS |
| GmTCP8 | MEEDDDLHHHHHRQNNFPFQLLEKKEDQETASCSTSSLYPSLAISAEPSTSNSTRSNQLAVAAAAEAPNSKKPPPKRTSTKDRHTKVDGRGRRIRMPALCAARVFQLTRELGHKSDGETIEWLLQQAEPAVIAATGTGTIPANFTSLNISLRSSGSSMSVPSQLRSSYFNPNFSLQQHQRRTFFPGIAFSSDNTNNNTSTLLNFQSNTLNTTMLQQQAKPELSDGGVPSSLDISDTNTEDTTTTTTTTTNLSRKRRPATTEQDLSSSTQQHQMGSYLLQSSAGAIPASHAANIWMVANSNSNQVMSGDPIWTFPPVNNSALYRGTMSSGLHFMNFPTPVALLPSQQLGSSGNIGAVGGSSHNNHNNNINEGHLSMLAGLSPYRPVIGVSESQASGSQSHRGSGTDDRHDNSSSHHS |
| GmTCP9 | MDQDDDEAGTTNLSTSDADVAEKETTNNLNGVPKQNNAFEETTGFQVLPLKKEEPTYSDPDTEIVPVKLPKRSSTKDRHTKVEGRGRRIRIPATCAARIFQLTRELGHKSDGETVRWLLEHAEPAIIEATGTGTVPAIAVSVGGALKIPTTSSNSNEEAAAAAASSNKKRKRPSNSEFVDINININDAVSKSSGLAPVHVPQGLVPVWAVSNPSMVIPANTFWMIPQAATTPNPSGGVAGPASQQPQLWALSPSVFNVAARPISPLVTTNIPEARPVMTACSNGSNSAVSTSTVGAKLATKSSMAPSVSSSGTKSGKTQMLRDFSLEICDKQELQLLGRSGTHAQS |
| GmTCP11 | MEEEDEIQAQKVRRRRGSDGESTNRFHSSWHHHHHHSSRIIRVSRASGGKDRHSKVMTSKGLRDRRVRLSVTTAIQFYDLQDRLGYDQPSKAVEWLIKSASDSISELPSLNNFPDTPKSDDHEKRANTDVAAGAVDGENYENENGNNNNQSQNMSLSKSGCSSTSETSKGSGLSLSRSDIRVKARERARERTTKEKEKEKNESHHHHSNVVVPHHRHHHHHNPVSQTASFTELLTGGIPKGCDDEEPNNMMMFNKEAAAASSGRQQWCSSSSAAAAAPMDYFLGPSSSRTTTTPPHNHHHHHHQYSSSGGFSLAQIQLGHSLPEAMNHNVSAFNNNVSGENQNHSSSDPHLQQQQHFSFIPDHLMSAVVTSSSHHQPSGSDYNLNFTMSSGLAGYNRGTLQSNSPSLLPHLQRFSPLDGSTVPFFIGAAPSAAVAAPAMENNTTTTNNHHHHQFSSGFDGSRLQLYYGHSDQKGKAKN |
| GmTCP12 | MEGGSDDFHYHHHLHNHNHHRQNFPFQLLEKKEDPQDPSSCSTSSPYPSLAISAEPSTSNNNNQLLPTPSSEPSPKKPPPKRTSTKDRHTKVDGRGRRIRMPALCAARVFQLTRELGHKSDGETIEWLLQQAEPSVIAATGTGTIPANFTSLNISLRSSGSSMSVPSQLRSSYFHNSSFSLQPRRSLFLENTNTNTPNTTTTTLLNFQSNNNNNPIFQIQPKHELRESSSLDLSSENNNNNEEGNLGRKRRPLEQDLSSTMQHQVGIGNYLMQSSTTSGTIPASHHAQIPANIWMLANSNSHHNQGGGVSVSGGEAPPLWTFPGGVSNSALYRGTMSSGLHFMNFPTPMALLPGQQLGSSGIGGNMNEGHLNMLSGLNPYRTTVIGVSESQASESQQSHHGGSEDQHE |
| GmTCP13 | MMLTMMASSREGYQAKQEGDTTTNIDKFSKASSTTSRQWSAFRNPRIVRVSRSFGGKDRHSKVCTIRGLRDRRIRLSVPTAIQLYDLQDKLGLSQPSKVIDWLLEATKFDIDKLPPLQFPQGFGQFHHHPQTLFPFHESSAASHQLSLGPFSDASSTFVRDGGIQNLMAKSRYWENINSMSRLRGNKEAEKGKWIKTSEEENHDQDGVVGGYNNLQVSTQRLFPMGSSTTTTTHSLLPGLLNNSMPYNAYSSEPSSLSLSQFGSHGLFPSQQVDPNPSSGNNGVQFQSSLSLPSSGSQLLFGSSSATPSMFTTYAPFIAPSVDPDPRQFNHIHQFLNSGSQILPPHPLIPSLHHPFNSQIRPFPAALFSSKLLDSDSSNHSQQDHKGSTS |
| GmTCP14 | MGELQHHQQQAAAAAAPSRAAMRGVGGGEIVEVQGGHIVRSTGRKDRHSKVCTAKGPRDRRVRLAAHTAIQFYDVQDRLGYDRPSKAVDWLIKKAKAAIDQLAELPPWNPTATSMQPRQLPQEIVHCENKSLAMDDSMTAAFSSRGESNAFASHQQVEDNENANNISSSSGNKYNTGSGFLPASLDTDNIAETIKTFFPVEATSTSFQSYPPAPPDLGHQDLRLSLQSFQDPIMLHHQPHSHHQPVLFAGTAAAALDFDGGSGWSEHLHQHQQQNHHSEEQRLLYAGGNSGHGGGFVFNTPAPVQVPAFGQFFSQRGPLQSSNTPSIRAWIDPSVDHHHHHHHYLSQLIHQGSVAGGGGFSGFRIPARIQGEEEHDGVSDKPSSASSDSRH |
| GmTCP15 | MRTNVGDIVQVEGGHIVRSTGRKDRHSKVYTSKGPRDRRVRLSAHTAIEFYDVQDRLGYDRPSKAVDWLIKKAKTSIDKLAELPPWHPPTNHEEEEQNDAAGSSGVIAIEQQQQQQQQQQQQQQQQQQQQQHQQQQSESCGYNFQLQRQLGAFISTHVDTDHINFQTNNNNSSEDLGLSLHCFQDHPGLIQWQSQQEGANQTPPSNEHQIQQTPFAGSTPVGFENHYQRSVTWNNEATTTDHVNRLGFLFNSQPYASAYAQSGGTLQSSFSFPMTSSSELHRPQPVNQPSSIFGSRFVSDGLAGFCIPDRIQGVEENHGVASNRPSSSPSSIH |
| GmTCP16 | MADEASGDVDLRLGEPKAPKEEPETEEGSAIRVMPVAVHVPAGMSMSMSKALAQAQAQAQAQAQPQKRASTKDRHTKVEGRGRRIRMPATCAARIFQLTRELGHKSDGETIRWLLEHAEPAIIAATGTGTVPAIAMSVNGTLKIPTTSPSDQEPGDQPERKKRKRPANSAYVDINDAAVSISTGLASSNNNNQNAATTTTMTIPQHTAIPLPQGMVPVWAIPSNAVVPAAGAFFVVPQTASFQHQPQFFTIARPISAFVSSLVTPVQPQPTSITSSSNANASSTAPSSKSAARATVMAPTTTTATTTTTQMLRDFSLEIYDKQELQFMSRASSKH |
| GmTCP17 | MEGGEDLHHHHHHHHRHNNFPFQLLEKKEDQETASCSTSSLYPSLEISTKPSMSNSTRSNQLVVVAMVEAPNSKKPPPKRTSTKDRHMKLTRELCHKSDGETIEWLLQQVEPAVIAATGTGIIPANFTSLNISLRSSGSSITFFPGIAFSSDNTNNNTSTLLNFQSNTLNTTMLQQQAMPELSDGGVSSSLDILDTIV |
| GmTCP18 | MEEEDEILQARACKLPRRRGSDGESTNSRFHSSSWHHHNSSRIIRVSRASGGKDRHSKVMTSKGLRDRRVRLSVNTAIQFYDLQDRLGYDQPSKAVEWLIKSASDAISELPSLNNFPDTPQSDDHEKRENTADVAVGVVDGENGNGQNLSLAKSACSSNSETSKGSGLSLSRSDIRVKARERARERTTKEKEKEKEKNDESHRHHHNVVVVPHHNHNVNLVSQTASFTELLTGSDPNKGCDEANNNMMFNKAAGRLQWCSSTTPMDYFLGLSSSRTTTHNHNHLHHHQYSSSSSSGFSLAQIQLGHSLPEAMNHVSAFNNNVSGDNHNHSSSDPHLQQQHLSLIPDHLMSAVVTSSAASSHHHHHHQPSGNDYNLNSTMSSGLAGYNRGTLQSNSPSLLPHSQRFSPIDGSTVPFFIGAASSAAAVAAPAMENNNNNNHHQHQFSSVFDGSRLQLYYGHSDQKGKAKN |
| GmTCP19 | MIKSQKEADFQLKQEGLSQSINDPEKAKATSSSVAQWPRLKDPRIVRVSRAFGGKDRHSKVCTIRGLRDRRVRLSVPTAIQLYDLQDRLGLSQPSKVVDWLLNAAKHEIDELPPLPIIPSVNNFTLGYPSAVTSNEATTSNSQPNEQLLNINRSIQWEDSNHNSTWKLKAKEVSGEMVSTDKPNWISRSQEDKQGSNNQGPSTHVIPNINLLPRANLNHPSFLGLLNTMPHGYRWEHSAGDVSHPLGNNELSNLTDIHNNINVVPFPTSSLSLSTGNSQIMLCPPGATQPYFPSHVTAMDMDPRQINHYQMLSSGSENPLANSLNHSFSLAMMAHKPLLSPNSSKSPSHKDQDFP |
| GmTCP20 | MFPSTNYTSSGSYPRFPSSSSSSTSPYPSFTLLHPENSSSSNTFLCDPLALTYIPSHYHAPIIPETLANWAVADCAILNQDLGGALYGITNKPEKKATKKDRHSKIHTSQGLRDRRVRLSIEIARKFFDLQDMLGFDKASNTLDWLFTKSKKAIKELTRSKHSADSFEFSSSSDGEVVSTIHQDLHQQQGVDLEEGKLKEPAAYCVKAKMKESREKARARARQRTSSKVLCNISSEGKVQDLKKKCPATENPQILNQLRSPLQPPHPQDVGGEVPRDDDFNVIEESIVIRRKLKHTLMSSIHHQNAVIPKEASVNNSDYHSFPNLSPNWEANNNGANGRSTFCAIASMNLSTGLQIFGKSWEECTNPHPS |
| GmTCP21 | MEEECVHQQNVAGNKSNFPLQLLKKKDEGPPPSPPPSAAKAKRPPTKDRHTKVEGRGRRIRMPAACAARVFQLTRELGHKSDGETIEWLLREAEPAVIAATGTGTIPANFTSLNISLRSSGSSLSSPSHYFLNRRNNLYNHNNHPFVTTTPIFSSSESCFKSYPSFSFAEDSKRSSFSDSSFANNYYSRKRRHEVISHVGSCCSSQMCTDSVPAMMGGADSVWAIPSVASSSASGIPFMNFASLLTGQHVGGATLSSETNFNVLATLNAYSGQQHQHRHEGEETHY |
| GmTCP22 | MSNSDGATNAVPPNDANNNTIVEYQQRTTTTTTAAAAALAVKKPPSKDRHSKVDGRGRRIRMPIICAARVFQLTRELGHKSDGQTIEWLLRQAEPSIIAATGSGTTPASFSTVSDHKPLLPPPTPFILGKRIRPDDDSAAKDDAVSLVAPPTPPALWALPPRPDFGQVWSFAAAPELVSVSPQNSMFHHHHQHHQQQQQQAAMGEASAARLGNYLPGHLNLLASLSGGHGNSGRRDDEPR |
| GmTCP23 | MSNSDGATNAVPPNDANNNTIVEYQQRTTTTTAAAAALAVKKPPSKDRHSKVDGRGRRIRMPIICAARVFQLTRELGHKSDGQTIEWLLRQAEPSIIAATGSGTTPASFSTVSDHKPLLPPPTPFILGKRIRPDDDSAAKDDAVSLVAPPTPPALWALPPRPDFGQVWSFAAAPELVSVSPQNSMFHHHHQHHQQQQQQAAMGEASAARLGNYLPGHLNLLASLSGGHGNSGRRDDEPR |
| GmTCP24 | MTSQVVLHNNFETQPSVTVTTATNTESPPTAPPSLAVVKRPSTKDRHTKVNGRGRRIRMPPLCAARIFQLTRELGHRSDGETIEWLLRHAEPSIIAATGSGTVPAAPVSSVAPSAPSSEPMVACPVTAVGAQGMFAMAPPQPQPNCRLDLSPPAGLEFAVNGYRHMPFTALLLQPATAEDNQNDESLGEQ |
| GmTCP25 | MEVNPTHDGGDDTSETPLPPMVDPSPSPSPQMKEEVMDAEQGGDVSLPMSVVPASLTVSTEAAAKRSSKDRHTKVEGRGRRIRMPATCAARIFQLTRELGHKSDGETIRWLLEHAEPAIIEATGTGTIPAIAVSVGGTLKIPTSSEARAQGEEDTPKKRRRRALKSEFINLNENQVSVSSGLAPIAQSSAYGSGGGGLVPLWHGNGAAAGPFFVFPNASNPPQYWAIPATAAPFFNVQARPISGFVSALQMQHDNHHHSLINAAASGSVKSSSTVSVTSSAASTQMLKDFSFEIYDKKELQFLGQPPQPSS |
| GmTCP26 | MFSSTYDSNFFPNFPLSTYPILPFLIESENDFASHTLLVEDPLVVPLTHDPPPFPEETVANFAVADHHCTAMLDNQHDAAANTNYGSHYGSSISNFLTHQKPAAATAKKDRHSKIHTSQGLRDRRVRLSSEIARKFFDLQDMLEFDKPSNTLEWLFTKSENAIKELARSKHSGSVSTGGDKSSRDPSGDSNNNFNNNKSMVGGGVGDGSKGRKLKWAQRGDACIQNKKESRERARARARERTCFKKCSSIRVQQQKDFDERCPATTNTTQMLHQLWSSIQPEPEEPRARWVQPYYNPYFVDDNEAPRDHGFNVIEESIMIKRNMKPLSQPNLVIPREASFNNNEFPLLPYSTTPNWESTNGAVNYCGISTMNLSTCFMNP |
| GmTCP27 | MSNSDATTNGVSNGAIIDAQRQQALAVKKPPSKDRHSKVDGRGRRIRMPIICAARVFQLTRELGHKSDGQTIEWLLRQAEPSIIAATGSGTTPASFSSVSLSVRAAANSLSSPSSTSDHKPQLLSPTPFILGKRIRTDEDSSKDEAVSVGPSLVGPSTPPGLWALPARPDFGQIWSFAAAAAPEMVSVSQQQQASLFAHHHRQQQQQAIGEASAARVGNYLPGHLNLLASLSGGPGNSGRRDDEPR |
| GmTCP28 | MASETPITLKAINTKLPSLKAKKDRHTKVNGRERRVLLPPLCAARVFQLTHELGYKTHGETIEWLLRQAEPSIIAATGTGILPSSMVVSASTSTPSLCEDESTIGMHTNPMVVSTDNEIESKKEFLPLDLDSLANFDVEFSANEVAILQSLITKLG |
| GmTCP29 | MRTGVGDIVQVEGGHIVRSTGRKDRHSKVYTSKGPRDRRVRLSAHTAIEFYDVQDRLGYDRPSKAVDWLIKKAKTSIDRLAELPPWHPPTHEEEKQNDAAGSTPKAIGAFSSTHADTDPINFQTNNSSEDLGLSLHCFQDHPGLIHWQSQQTGAHQTPPSNEHQIRTLFAGSTPGGFENHYQRSVTWNNEATTTDHVNRLGFILNSQPFIGQGSTSAYAQSGGTLQSSFSFPMTSPSELHRPQPVNQPSIFDSRFVSDGLADFCIPDRIQGVEENHGVASNRPSSSPSSIH |
| GmTCP30 | MGEPQHPHQHHQQAAATPSRAAMRGGGGGEIVEVQGGHIVRSTGRKDRHSKVCTAKGPRDRRVRLAAHTAIQFYDVQDRLGYDRPSKAVDWLIKKAKAAIDQLAELPPWNPTATPMQPPSQEIVHRENKSLGMDDSLTAFSCRGESPAFAAATRDSEQFSHPQVEQNENANNISSSSISIKYNSGSGFLPASLDTDNIAETIKTFFPVEATTTSFQSYPPAPPDLGQQDLRLSLQSFQDP IMLHHQPQSHHEPVLFAGTAAAALGFDGGYGWSEHQHQNHHSEEQRLLYAGGNSGHGGGFVFNTPAPVPAFGQFFSQRGPLQSSNTPSIRAWIDPSVDHHHHHHHYLSQLIHQGSVAGGSGFSSGFSGFRIPARIQGEEEHDGVSDKPSSASSDSRH |
| GmTCP31 | MELSDLQNKKQSSSSSSTGATHQQQQQQQQSSSTHLVVPSPSNSSASLIDASLAIATRSDDSTKKTQQPSTNTKRSTKDRHTKVDGRGRRIRMPATCAARVFQLTRELGHKSDGETIEWLLQQAEPAIIAATGTGTIPANFSTLNVSLRSSGSTLSAPPSKSAPHTFHGALALAHHPYDEAFQHPALLGFHPHHHQQQLLSADQIAEALPSAGGGDSGGNYLRKRYREDLFKDDNINNTQ SQNDSGDADGDADGDGSSPKLPKEAGSGLLRPTNLLPATAMWASGPPGSTIWMLPVTAAAASSSSSSSSASSAASSESQMWAFPQSVSGFMPRFNLPVPGALEFQGARAGSLQLGPSMLMPPPPLPPQQQQQPSQHLGLAMSDSNLGMLAALNAYARAGLNINSDHHHHHHHHHHHQHQSQPSESGEDAPNSSQ |
| GmTCP32 | MGMKSTGGEIVQVQGGHIVRSTGRKDRHSKVYTAKGPRDRRVRLSAHTAIEFYDVQDRLGYDRPSKAVDWLIKKAKNAIDKLGELPPWHPTPNTAADAENNNNNNNAGSSDMAIAAEQSESSGYNFQLQRQLGEDHDNHHSAFIPSPIDTDAIAFFPTTTATSSINFQTYPPNIISRTNNSTEDLGLSLHSFQDPGLIHGQTQAGANQTQTPSNDQSLFSGSTQVGFEANYPRIVTWNSD ASIDMNRTGFMVNSPALLGQGGSAAFSQRGTLQSSFSPSLRPWSEIPMASSEHHKSQPIQQASIFGSRFLSDALPGFCIPARIQGEDGVGPDKPSSSSPNSHH |
| GmTCP33 | MVRASGGGGGEIVEVEGGHIVRSTGRKDRHSKVCTAKGPRDRRVRLSAHTAIQFYDVQDRLGYDRPSKAVDWLINKAKSAIDQLAHLPPWKPTLPAPQNDDESDKLNPNPNPIPIHHPDAEILHQFNENDTENLRCDGGGGSSYLPMDNDAIRSFFPTTTSSLVHFQSYPPDLLSRTSSQDLRLSLQSLQDPVLLHQNHHNNEHVLFAGTGFENMVAWNSSSNNNNNTASNDTCGGGGGGGGGGGGGGFVFNAPSTLTGPATVASPAVVFGHGLGQGQYFTQRGPLQSSNSPSVRAWIDAPSFVAAAADHRHHHHHYLSPAAAAAALVYQSAPSTAAFAAAPGGFSGFRVPAQIQGEEEHDGGVSDKPSSASSDSHR |
| GmTCP34 | MFPYSSNPSPSFPSSSSSYPPFPFLNLENASASNTLLHDALSVPYIPTHHNPSSIQEALNNFPVTDHNCGGGGGSGSAMTKPDPNGGGAPQYGISCFLTKKPPKKDRHSKIYTSQGLRDRRVRLSIEIARKFFDLQDMLGFDKASNTLEWLFNKSKRAIKELARSKHSNNNSDEGAKSFSSSSDCEDGCEVVSEIKHKQQQLITCDAAATTPHNLQQGLDSGDNKSPTTLMAAKERMKLKRTQKEPAKMKESREKARARARERTSNKIIMCNVNTNNTGGKVLQDLKKKCPAIENNHTQIMHQSRSSPTHHPHHLVGNETPRDDFNVIEESIVIKRKLKPSLMSSSDHHHHHHQNLVMPKEPPSFNNNNSEYHPFSNLSPNWDANGSTGRSNFCAIASMNLSTGLQIFGKSWEECINPR |
| GmTCP35 | MTSQVVLHNNFETKPSATVTTIVESPSVAPPSLAVAKRPSTKDRHTKVNGRGRRVRMPPLCAARIFQLTRELGHRSDGETIEWLLRHAETSIIAATGSGTVPAAPVSSVGPSAPSSEPMVACPVTAVGAQGMFAVPPQPSCRLDLSPPPGMEFAVNGYRHMPFTALLLQPATADENQNDESLGEQ |
| GmTCP36 | MEEDEIQACKFPRVGNGRSSDQYQEEEEEGGDLRLRKGVGVGGGGGGGGSDASTNHFHQSSWHHSSRIIRVSRASGGKDRHSKVMTSKGLRDRRVRLSVTTAIQFYDLQDRLGYDQPSKAVEWLIKAASEAISELPSLNNPFPDTPKQLSDKKRPTSGGGQQQQQIQQGHFDDADGDTSYPQNQSQNLSLSKSACSSTSETSKGSGLSLSRSEIRVNRVKARERARERAAKEKEKEKEKEKEKEKESESSIAHHHHHHHVNNNNHMSHTASFTELLTGGIGSTVTVNPNTTTTTTTTTTSPNGSSVHQIHDGHDHETNLFNKGRQQQQQHHWSQTVTPMDYFSTGLLVGPSSSSARTQQHQHQSSSAHFQLGHAHALPISPFSGENHSEQLQHFSFMPDHLNNIPAHHVVTSSSASHQPNGGDNNYNLNFSISTSTGLAGYNNRGTLQSNSPSSSFLPHHLQRFQPLDGSSNLPFFIGAAAPSSAPPTMDNNNNNNSNNHHHLQFSPVFDGRLQLCYGDGTRHSDHKGKGKN |
| GmTCP37 | MVRGSGGEIVQVEGGHIVRSTGRKDRHSKVCTAKGPRDRRVRLSAHTAIQFYDVQDRLGYDRPSKAVDWLINKAKSSIDQLAHLPPWKPTLPSPAPPHLNDDHSDKPNPNPNPNPNPNPNPNHSPDAQILNQFDHDDGAGAGSSYLPMDNDAIRSFFPTTTSSLVQFQSYPPDLLSRTSSQDLRLSLQSLQDPILLHHNHNNNEHVLFAGTAFDNMVAWNNNSNNNHNNNNHNNNNNNNNNNNTASNDTCGGGGGGGGGGGGGGGGFVFNAPSTLPGPATVASPTVVFGHGYGQGQYFSQRGPLQSSNSPSVRAWIDAPSFVAAAADHRHHHYLSPAAAAAALVYQSAPPTAAFTAAPGGFSGFRVPARIQGEEEHDGGMSDKPSSASSDSRR |
| GmTCP38 | MGMKSTGGEIVQVQGGHIVRSTGRKDRHSKVYTAKGPRDRRVRLSAHTAIQFYDVQDRLGYDRPSKAVDWLIKKAKNAIDKLAELPPWHPNPNTTAADAENNNNNNAGSSDMAIAEQSESSGYNFQLQRQLGEDHDNHHHSAFIPSPIDTDAIAFFPTTTATSSINFQTYPPDIISRTNNSTEDLGLSLHSFQDPGLIHHGQSQAGANQTPSNDQTLFSGSTQVGFEANYPRIVTWNSDASTDMNRTGFMVNSPSALLGQGGSAAYSQRGTLQSSFSPSLRPWSDIPMASSEHHHKSQQASIFGSRFLSDALPGFCIPARIQGEDEGHGVGNDRPSSASPNSHH |
| GmTCP39 | MEEDEIQACKFPRVGNGRSSDQYQEEEEEGDLRRKGVGGGGGSDASTNHFHQSSWHYSSRIIRVSRASGGKDRHSKVMTSKGLRDRRVRLSVTTAIQFYDLQDRLGYDQPSKAVEWLIKAASEAISELPSLNNPFPDTPKQPSDEKRPTSGGGQQQQQQGFDDADGDTNYQQNQSQNLSLSKSACSSTSETSKGSGLSLSRSEIRVNRVKARERARERAAKEKEKEKEKESDFNIAHHHHHHNVNNMSHTASFTELLTGGIGSTVTVNPNTTTTTTTTATSPNGSSVHQIHEGHDHEANLFNKGSQQQQQQQHHHWSQTVTPMDYFSAGLLVGPSSSASTQQHQSSGQFQLGHGHAHAHALSISPFSGENHSEQMQHFSFMPDHLNNMPSSAASASQHSEGDNNNYNLNFSISSSGLAGYNRGTLQSNSPSSSFLPLLQRFQPLVDGSSNLPFFIGAAPPSSAPPTMDNNNSNNSNNHHHLQFSPIFDGRLQLCYGDGTRQHSDHKGKGKN |
| GmTCP40 | MKKLTGGASSPASSSSLAIPTANPKRSTKDRHTKVDGRGRRIRMPATCAARVFQLTRELGHKSDGETIEWLLQQAEPAIIAATGTGTIPANFSSLNVSLRSSGSTLSAPPSKSAPHTFHGALALAHHPYEEAFQHPALLGFHPHAHQPQQLLSADHIPESLPSGAGDSGDNYLRKRYREDLFKDDNINNNNTQSQNESGDGDGSSPKLPKQQSEAGSGLLRPSNLLPATAMWAVAPSPASGPPGSTIWMLPVTAGASSSASSETQMWPFPQSVSGFMPRFNLPVPGALEFQGARAGSLQLGSMSMPQQQHLGLAMSDSNLGMLAALNAYTRASLNVNSDHHQHQHQSQPSESGEDDPNSSQ |
| GmTCP41 | MDPKGSKQQPQQSQEVVPNFLSLPQQQQGNTNNNNMGENKPAEVKDFQIVVAENKEESKKQQQQLAPKRSSNKDRHTKVEGRGRRIRMPALCAARIFQLTRELGHKSDGETIQWLLQQAEPSIIAATGTGTIPASALAAAGNSLSPQAASLSSSLHQHQQKIDELGGSGGSSSRASWQMVGGNLGRPHLGVGVATAAGLWPPHVSGFGFQTPPTTTTPTTTTSSSGPSNATLATESSNYLQKIAFPGFDLPTSATNMMGHMSFTSILGGGGGGGAQHMPGLELGLSQDGHIGVLNQQALNQIYQQMNQAGRVHHHQHQHHHQHHQQQQHHQQTPAKDDSQGSGGQ |
| GmTCP42 | MMIESSSKVHGANNQQEGDDNNIKIEKLSKAPSTSSSSRSWSAFRNPRIVRVSRSLGGKDRHSKVCTIRGLRDRRIRLSVPTAIELYDLQEKLGLNQPSKVVDWLLETTKSDIDKLPPLQFPHCFAHHHQQTLLPGTSQFSLGGFYDAAANGGNENHLMATKSTRYWDIDSHSLNNGKDLVESVSNSLKGKSWIKTNEQENHQDGGIGGSSSTHNNREEYSSAHYKLFPIGTTTNNSYLPGLLNNGMTHNSSNHHSEPSSLSLSHFGSHGLFPSSHNAHPSSCSGVPFSSCNFSAASSGPQLLFCPPTSATPASAFYSVESDPRQSNNNVQIFSSSSQVMMPHPLIQSLHSTNSTLNRRIPTSFSSKLLDSDNNNDRSQPNKGSSTTRS |
| GmTCP43 | MEGGDDLQHHHRQNNFPFQLLEKKEDQETASCSTSSPYPSLAISAEPSMSNSTRSNQLSAAEASAEAANSKKPPPKRTSTKDRHTKVDGRGRRIRMPALCAARVFQLTRELGHKSDGETIEWLLQQAEPAVIAATGTGTIPANFTSLNISLRSSGSSMSVPSQLRSSYFNPNFSLQQQQRRTFFPGIALSSEHNTTTNNTSTLVNFQQSNNLTTTMLQQQAKPELRDGLPSSLDISDTRTNTEDPSRKRRPTTEQQDLSPTTQQQHHQMGSYLLQSSAGAIPASHAANIWMVANSNSNQVMSGDPMWTFPPVNNSALYRGTMSSGLHFMNFPTPMALLPSQQLGSAGNIGAVHGGSSNNNNHNNNMNEAHLSMLAGLSPYRPVIGVSESQPSGSQSHRGGADDRHDNSSSHHS |
| GmTCP44 | MYSSNTFLNGNDLISYPNQPFCFRPFSFESNPTYNFSKEEANSNYALPPPPPPPPLSFFQSPFDENIFLEHHHHDLLLLHQYSLTDSGVSKNLEVVAEISPIPCPEQGGIAMEHTPRKRSSKRDRHSKINTARGLRDRRMRLSLEVAKRFFGLQDMLGFDKASKTVEWLLNQAKGEIKQLAREKTSVVGGGGKSASSTSECEGVSSLDEVAVSTGGVNNEEQERETVPNMMKRRKSKVCRKSAFNAIDKESREKARERARERTREKMRTRRVLVSDASNLNRLSSWNPFETVEDSAGTTHHPSLDVHLHEADQEPISSHNVKEHNLGEDMAHEDNSLALMNKWSPTMLFNSLHNSGILQEHQFAEFQSLGKQWEAYSNQI |
| GmTCP45 | MASNHEMDQDDDGGATDLSTSDAETTTTNLNGVPKQHNAFEEETTGFQVLPLKKEEPTYSDPNMGITPVAMQVPVKPAKRSSTKDRHTKVEGRGRRIRIPATCAARIFQLTRELGHKSDGETVRWLLEHAEPAIIEATGTGTVPAIAVSVGGTLKIPTTSSNEKEAVADASNKKRKRPSNSEFVDININDSVSQSSGLAPVHVAHGLVPVWAVTNPSMVVPANAFWMTPNPPMGPANQQHQLWALSPSVFNVAARPISPLVTTNIPEAWPIMTACSNDSNSAVSTSTVGAKVATESSMAPSVSSSGTKSARTRLLRDFSLQIYDKQELQFLGRSGTHAQS |
| GmTCP46 | MEEQRVHHDENVTRKPNFPLQLLEKKDEGPPPPPSAAKAKRAPTKDRHTKVEGRGRRIRMPAACAARVFQLTRELGHKSDGETIEWLLREAEPAVISATGTGTIPANFTSLNVSLRSSGSSQSHYFLNRHNNLHNHNTNNYPFFTTTTPPEFSSSGSYFKSHPSFSLMQNSRRFSRKRRHEVISHVGNYCLSQMCTDSVPAMMSGADSVWAIPSVASSSASGIHFTNFASLLTGQHVGGAMVSSETNFNVLATLNAYSGQQPHHRHEGEETNN |
| GmTCP47 | MVPWNRILTITKWKDKTQHLATNNERHKIMIKSPKEADFPLKQEGHSQSITDPEKAKASASSVAQWPRLKDPRIVRVSRAFGGKDRHSKVFTIRGLRDRRVRLSVPTAIQLYDLQDRLGLSQPSKVVDWLLDAAKHEIDELPPLPVVPSVNNFTLGHPSAVTSNEATTSNSQPNEQLLNINRSIQWEGSNQNSTWKLKPKEVSREMISTDKPNWISRSEEDKQGSNNEGPSTRVIPNINLLPRANLNHPSFLGLLNTMPHGYQWEPSAGDVSHQLGNNGFANQTDMHSINVVPFPTSTLSLSTGNSQILLCPPGTAQPYFPSHVTMDMDPRQINHYQMLSSGSQNPLANYLNHSFSLAMMTHKPLHSPNSSKSPSHKDQDFPSN |
| GmTCP48 | MFPSTNYTSSGSYPRFPSSSSSTSPYPSFTLLHPENSSSSNNTFLHDPLALTYIPSHYHAPIPETLANWAVADCAILNQDLGGALYGITKKPMKKATKKDRHSKIHTSQGLRDRRVRLSIEIARKFFDLQDMLGFDKASNTLDWLFTKSKKAIKELTRSKHSVESFEFSSSSEGEVVSTIQQQDLQQQHGINLENGKLKEPAAYCVKAKMKESREKARARAREKTSSKVLCNTSGEGKVQELKKKCPATENPQILNQLRSTLQPPHPQNVGGEVPRDDDFNVIEESIVIRRKLKHTLMSSNIHHQNAVIPKEASVNNSDYHSFPNLSPNWEANNGANGRSTFCAIASMNLSTGLQIFGKSWEECTNPHPS |
| GmTCP49 | MINSTKETDFPLKQEGLSSSDHEKAKAPSSSSSSQWLKLKDPRIVRVSRAFGGKDRHSKVCTIRGLRDRRVRLSVPTAIHLYDLQDRLGLNQPSKVVDWLLNAAKHEIDELPPLPIPPGNFTLGYPSLVSCNEVSTSREGSGQNTLLWKPKPGEIMVSDHDKANWMNRREGDDDDDNNHNNSNGDKQGSNNNCHGGALVLPNNLLPTRPNHPSFLGLMNTMPSLGYQWEPNSSAADVNVQWQNHGFFNQTDVHSIDVVPFPSTLALSTGNSTSTTTTSQILVCPPPPGATTQPYFPSSHFATMEMNARQINHYQMLSSSSHQNLLANSLNHPSSQQLMSQSGKAPFSLRIRPKLFHSPNSSESHSQKDQDFPSK |
| GmTCP50 | MFPYSSNPYPSFPSSSSSSYPPFPFLNLENASASNTLLHDPLSVPYIPTHHHNPSSIQEALNNLPVTDHNCGGGGGSAMTKPDSNGGGAPHYGISCFLTKKPPKKDRHSKIYTSQGLRDRRVRLSIAIARKFFDLQDMLGFDKASNTLEWLFNKSKRAIKELARSKHSNNNSDEGAKSFSSSSDCDDDCEVVSEIKQKQQQLITCDAATTQHNLQQGLDSGDNKSPTTLVAARERMKLKR TQKEPAKIKESREKARARARERTSNKIIMCNVNTNNNNTGRRVQQQDLKKKCPAIENNHSQIMHQSSSSPTHNLVGNETPRDDFNVIEESIVIKRKLKPSLMSSSHHHHHHHQSLMIPKEPPSFNNCEYHPFSNLSPNWDANGSTGRSNFCAVASMNLSTGLQIFGKSWEECINPR |
| GmTCP51 | MDPKGSKQQQPQEVVPKFLSLPQHHYQQQGNSNNNNMGENKPSEVKDFQIVVAAEKDESKKQQQLAPKRSSNKDRHTKVEGRGRRIRMPALCAARIFQLTRELGHKSDGETIQWLLQQAEPSIIAATGTGTIPASALAAAGNSLSPQGSSLSSALHHQHQKIDELGCGSGGSSRASWQMVGGNLGRPHLGVATTGLWPPHVSGFGFQTATTTTTTTSSGPSNATLATESSNYLQKIAFPGFDLPTSATNMGHMSFTSILGGAGSQQMPGLELGLSQDGHIGVLNPQALNQIYQQMNHQAQAGRVHQQHQHQQQQHQQTPAKDDSQGSGGQ |
| GmTCP52 | MSNSDGTTNGAITEAQRQQQQQGGKALAVKKPPSKDRHSKVDGRGRRIRMPIICAARVFQLTRELGHKSDGQTIEWLLRQAEPSVIAATGTGTIPASFSSVSLSSPSSTSDYKPQLLAPTPFILGKRIRTDEDSSKDEAVSVGPSLVGPYAPPGPGVVPAWPDFGQIWSFVAAAAAPEMVSVPQQQQQQASLFAHHHRQQQQLAIGEALAARVGNYLPGHLNLLASLSGGPGNSGRRDDEPR |
| GmTCP53 | MSSSTYDSSNLFPNFPSSSYPILPFLIDPENDFASHTLLDDHPLVVPAGLIHDPPLLPEETVANFAVAADCTAAMLEHDAANTNYGSHYGSSVSNFLTQKPAAATAKKDRHSKIHTSQGLRDRRVRLSSEIARKFFDLQDMLEFDKPSNTFDWLFTKSENAIKELARSKHSGSVSRGDIKYSRYPSVDSNNNNKSLVDGGDASRGRKLKWAQRDDVCVQNKKESRERARARARERTCYKMCSCSRRVQQKDSDERFPATTNTQMLHQLRSSIQPELEDQPRARWVQPYYNNPYFIDNEAPRNGFNVIEESIMIKRNMKPSSHQNLVIPRDASFNNNEFPLLPYSSTTPNWDSTNGAVNFGGISTMNLSTCFMNPW |
| GmTCP54 | MEANPTHDGDDTSETPPPPMVDPSPSPPPQMKEEVMDAEQGGDTSLPMTVVPAHLPVSKAAAKRPSKDRHTKVEGRGRRIRMPATCAARIFQLTRELGHKSDGETIRWLLEHAEPAIIEATGTGTIPAIAVSVGGTLKIPTSSAARPEGEVDTPKKRRRRASNSEFIDVNENQVSVSSGLAPIAQSAYGSGVGGGLVPLWHGNAAASGPFFMFPNASNPPQYWAIPATAAPFFNVQARPISGFVSALQMQHDNHHSLNGAASDSVNSSSTVGSTMSTVTVTTSSGSGSASGSSAATTTMLRDFSLEIYDKKELQFLGQPPPSSKP |
