# Supplementary material for: Genome-Wide Identification and Expression Analysis of TCP Transcription Factors Responding to Multiple Stresses in Arachis hypogaea L
Source: Int J Mol Sci. 2025 Jan 26;26(3):1069. doi: 10.3390/ijms26031069 (PMC11816611; doi:10.3390/ijms26031069)
Supplement: Supplementary file 1 [file ijms-26-01069-s001.zip › Supplementary Table/Supplementary Table S3.docx]

Supplementary Table S3 A list of primers

| Number | Primer name | Primer sequence (5′-3′) |
| --- | --- | --- |
| 1 | AhTCP2-F | TGATGATGTGGCAACAAGTG |
| 2 | AhTCP2-R | CAAGGGAAGAAGGAGGATTC |
| 3 | AhTCP6-F | CCATTCGTCTTCACACTTGC |
| 4 | AhTCP6-R | CTTGAAATCATCTCGTGAGG |
| 5 | AhTCP7-F | ACACTGATATAGCTGATACC |
| 6 | AhTCP7-R | TGGTGATGGTGGTGGTGGTGGT |
| 7 | AhTCP9-F | CACCGCTACACCGTACCTTA |
| 8 | AhTCP9-R | AATTCAAGACAGCAGAGTTG |
| 9 | AhTCP21-F | CCAAACAGATTTACATGGTG |
| 10 | AhTCP21-R | TGTTGTTGTCGCGGTGGTCGT |
| 11 | AhTCP30-F | CATCAGCAATTCCTTCAGAT |
| 12 | AhTCP30-R | AGTACCTTGATGTTGATTCG |
| 13 | AhTCP31-F | GTCTTTCTTTCCGATGGTGG |
| 14 | AhTCP31-R | TGGTGATGGTGGTGGTTGTGG |
| 15 | AhTCP33-F | TTAGCCACGCAGCCAGCTTC |
| 16 | AhTCP33-R | GCAGCAGCAGCAGAGTTGTT |
| 17 | AhTCP50-F | GAATCATCATCATGATGGGG |
| 18 | AhTCP50-R | ACTGTGTTGTGGCGGTGGTC |
| 19 | AhTCP25-F | GGGATATAATTCTCCTTCAGG |
| 20 | AhTCP25-R | GAATGGGGATCCAATAACTG |
| 21 | AhTCP36-F | AATGTTGTGCCTTTTCCATC |
| 22 | AhTCP36-R | CTGATGATGATGATTGATTTG |
| 23 | Actin-F | GAGGAGAAGCAGAAGCAAGTTG |
| 24 | Actin-R | AGACAGCATATCGGCACTCATC |
