# Supplementary material for: Genome-Wide Identification and Expression Analysis of TCP Transcription Factors Responding to Multiple Stresses in Arachis hypogaea L
Source: Int J Mol Sci. 2025 Jan 26;26(3):1069. doi: 10.3390/ijms26031069 (PMC11816611; doi:10.3390/ijms26031069)
Supplement: Supplementary file 1 [file ijms-26-01069-s001.zip › Supplementary Table/Supplementary Table S1 .docx]

Table S1 TCP members in Arachis hypogaea and their physicochemical properties.

| Gene ID | Gene ID | Number of Amino Acids | Molecular Weight(Da) | Theoretical pI | Grand Average of Hydropathicity | Subcellular localization prediction |
| --- | --- | --- | --- | --- | --- | --- |
| AhTCP1 | AH01G29310.1 | 231 | 25226.89 | 7.76 | -0.05 | chloroplast |
| AhTCP2 | AH01G32350.1 | 477 | 53168.1 | 7.03 | -0.99 | nucleus |
| AhTCP3 | AH01G33150.2 | 346 | 37848.79 | 8.6 | -0.63 | nucleus |
| AhTCP4 | AH01G34180.1 | 438 | 47452.7 | 7.15 | -0.82 | nucleus |
| AhTCP5 | AH03G00970.1 | 138 | 14934.79 | 9.51 | -0.54 | nucleus |
| AhTCP6 | AH03G15800.1 | 340 | 37930.69 | 6.17 | -0.82 | nucleus |
| AhTCP7 | AH03G18060.2 | 465 | 50201.9 | 6.75 | -0.7 | nucleus |
| AhTCP8 | AH03G19250.1 | 126 | 13066.54 | 4.99 | -0.19 | cytoskeleton |
| AhTCP9 | AH03G42230.1 | 392 | 40876.14 | 8.97 | -0.58 | nucleus |
| AhTCP10 | AH03G48440.1 | 462 | 51237.17 | 8.81 | -0.98 | nucleus |
| AhTCP11 | AH04G02510.1 | 496 | 51387.48 | 6.8 | -0.61 | nucleus |
| AhTCP12 | AH05G14810.1 | 259 | 29145.07 | 6.44 | -0.78 | nucleus |
| AhTCP13 | AH05G17680.1 | 454 | 47226.02 | 6.43 | -0.56 | nucleus |
| AhTCP14 | AH06G24470.1 | 349 | 37950.51 | 9.46 | -0.62 | chloroplast |
| AhTCP15 | AH08G14340.1 | 187 | 20085.43 | 4.76 | -0.38 | nucleus |
| AhTCP16 | AH08G18600.1 | 352 | 38532.98 | 6.14 | -0.84 | nucleus |
| AhTCP17 | AH09G25400.1 | 409 | 43554.38 | 5.38 | -0.51 | nucleus |
| AhTCP18 | AH09G26170.1 | 412 | 45870.65 | 6.43 | -0.76 | nucleus |
| AhTCP19 | AH09G30820.1 | 266 | 27818.06 | 9.72 | -0.41 | nucleus |
| AhTCP20 | AH10G23200.1 | 430 | 47648.74 | 9.33 | -0.7 | nucleus |
| AhTCP21 | AH10G25780.1 | 325 | 35481.5 | 7.84 | -0.56 | nucleus |
| AhTCP22 | AH10G27660.1 | 428 | 47028.01 | 9.26 | -0.87 | nucleus |
| AhTCP23 | AH11G07310.1 | 140 | 14133.79 | 5.6 | -0.18 | Mitochondria |
| AhTCP24 | AH11G23080.1 | 439 | 47566.8 | 7.15 | -0.83 | nucleus |
| AhTCP25 | AH11G24680.1 | 346 | 38014.05 | 7.95 | -0.61 | nucleus |
| AhTCP26 | AH12G08120.1 | 342 | 36938.58 | 5.54 | -0.19 | Cytoplasm |
| AhTCP27 | AH12G36570.1 | 201 | 21269.85 | 8.86 | -0.35 | nucleus |
| AhTCP28 | AH13G02850.1 | 139 | 15035.87 | 9.66 | -0.62 | nucleus |
| AhTCP29 | AH13G11230.1 | 170 | 17906.94 | 6.28 | -0.29 | nucleus |
| AhTCP30 | AH13G18380.1 | 337 | 37655.38 | 6.24 | -0.85 | nucleus |
| AhTCP31 | AH13G20740.2 | 466 | 50089.76 | 6.71 | -0.68 | nucleus |
| AhTCP32 | AH13G26220.1 | 469 | 52331.33 | 7.79 | -0.98 | nucleus |
| AhTCP33 | AH13G44850.2 | 398 | 41447.73 | 8.63 | -0.58 | nucleus |
| AhTCP34 | AH13G51140.1 | 461 | 51047.91 | 8.81 | -0.97 | nucleus |
| AhTCP35 | AH14G03310.1 | 495 | 51458.59 | 6.83 | -0.6 | nucleus |
| AhTCP36 | AH14G36430.1 | 380 | 42599.48 | 6.66 | -0.96 | nucleus |
| AhTCP37 | AH14G38100.1 | 467 | 51442.47 | 8.47 | -0.92 | nucleus |
| AhTCP38 | AH14G40990.1 | 140 | 14397.2 | 5.27 | -0.12 | Mitochondria |
| AhTCP39 | AH14G42340.1 | 456 | 50588.71 | 8.47 | -0.91 | nucleus |
| AhTCP40 | AH14G43910.1 | 381 | 42717.6 | 6.69 | -0.94 | nucleus |
| AhTCP41 | AH15G08070.1 | 265 | 29677.68 | 6.41 | -0.76 | nucleus |
| AhTCP42 | AH15G13900.1 | 452 | 47059.84 | 6.3 | -0.56 | nucleus |
| AhTCP43 | AH16G30140.1 | 304 | 32534.99 | 8.06 | -0.75 | nucleus |
| AhTCP44 | AH18G04790.1 | 188 | 20216.61 | 4.76 | -0.44 | nucleus |
| AhTCP45 | AH18G09970.2 | 352 | 38518.95 | 6.14 | -0.84 | nucleus |
| AhTCP46 | AH18G13740.1 | 368 | 40646.41 | 8.93 | -0.17 | chloroplast |
| AhTCP47 | AH19G37380.1 | 266 | 27813.04 | 9.51 | -0.4 | nucleus |
| AhTCP48 | AH19G41930.1 | 410 | 45771.44 | 6.46 | -0.79 | nucleus |
| AhTCP49 | AH19G42580.1 | 379 | 39716.06 | 5.82 | -0.5 | nucleus |
| AhTCP50 | AH20G33360.1 | 305 | 33336.01 | 8.3 | -0.57 | nucleus |
| AhTCP51 | AH20G35380.1 | 428 | 46989.88 | 9.2 | -0.87 | chloroplast |
